# Cerebellopontine Angle Epidermoid Cyst With Malignant Transformation Into Squamous Cell Carcinoma: An Unusual Complication of a Benign Intracranial Tumor

**DOI:** 10.7759/cureus.86466

**Published:** 2025-06-20

**Authors:** Dianela Gasca Saldaña, Ytel Jazmin Garcilazo Reyes, José Omar Navarro, Daniel Magos Rodríguez, Luis Felipe Arias Ruiz, Maria Lizeth Perez Diaz, Erick Gomez-Apo, Raymundo Hernandez Montes de Oca, Guillermo Axayacalt Gutierrez-Aceves, Ildefonso R De La Peña, Andrés Vega-Rosas

**Affiliations:** 1 Internal Medicine, Médica Sur, Mexico City, MEX; 2 Neuro-Oncology, Hospital Ángeles del Pedregal, Mexico City, MEX; 3 Neuro-Oncology, Hospital San Angel Inn Universidad, Centro Oncológico Internacional (COI), Mexico City, MEX; 4 Cancer Center, Médica Sur, Mexico City, MEX; 5 Neurological Surgery, National Cancer Institute, Mexico City, MEX; 6 Pathology, Médica Sur, Mexico City, MEX; 7 Neuropathology, Hospital General de México, Mexico City, MEX; 8 Radioneurosurgery, Instituto Nacional de Neurologia y Neurocirugia, Mexico City, MEX; 9 Oncology, Médica Sur, Mexico City, MEX; 10 Pain Management Center, Hospital Angeles Mocel, Mexico City, MEX

**Keywords:** benign lesion, epidermoid cyst, intracranial tumor, malignant transformation, squamous cell carcinoma

## Abstract

Epidermoid cysts (ECs) are typically located in the posterior cranial fossa, most commonly at the cerebellopontine angle (CPA). Although generally benign, the cyst’s epithelial lining can undergo malignant transformation into squamous cell carcinoma (SCC), a rare occurrence associated with poor prognosis. We present the case of a 55-year-old woman with a history of migraines who was incidentally diagnosed with a right CPA lesion consistent with an EC at the age of 30. Twenty-five years later, she developed progressive right-sided hearing loss, tinnitus, vertigo, headache, dysgeusia, and gait disturbances. Histopathological examination confirmed malignant transformation of the EC into SCC, supported by immunohistochemical markers including p63, p40, CK5/6, and D2-40. She was treated with fractionated stereotactic radiotherapy, carboplatin-paclitaxel chemotherapy, and the immune checkpoint inhibitor pembrolizumab, demonstrating good treatment tolerance and clinical stability.

## Introduction

Epidermoid cysts (ECs) are generally benign lesions of congenital origin. They predominantly affect middle-aged and elderly individuals (age range: 37-74 years; mean age: approximately 54), with a slightly higher incidence in females than in males. The specific incidence of cerebellopontine angle (CPA) ECs undergoing malignant transformation into squamous cell carcinoma (SCC) in Mexico is not documented in the national medical literature. Fewer than 100 such cases have been reported worldwide [1].

These cysts arise from aberrant migration of ectodermal elements during embryogenesis, which become trapped within neural tube tissues, forming ectodermal inclusion cysts [2]. A less common etiology involves secondary inoculation following surgical procedures or previous trauma [3,4].

ECs are most commonly located in the posterior fossa, particularly in the CPA region [5]. The CPA is a triangular space in the posterior cranial fossa, bounded superiorly by the tentorium, posteromedially by the brainstem, and posterolaterally by the petrous portion of the temporal bone. This region is clinically significant, as it contains the CPA cistern, which houses cranial nerves V, VI, VII, and VIII, along with the anterior inferior cerebellar artery.

Less frequent EC locations include the parapontine region, intraventricular system, parasellar area, and thoracic medulla. The incidence of ECs peaks in early adulthood, with the highest frequency occurring between the fourth and fifth decades of life. Clinical manifestations vary depending on the cyst’s location but are typically associated with cranial neuropathies, most commonly affecting cranial nerves V, VII, and VIII due to their proximity to the brainstem [5].

Although ECs are typically benign, the cyst epithelium can undergo malignant transformation into SCC, a rare event associated with poor prognosis [6]. The precise mechanisms of this transformation are not fully understood, but it is hypothesized to result from chronic inflammation caused by cyst contents and repeated ruptures, leading to cellular dysplasia and eventual neoplasia. While malignant transformation generally occurs at the primary lesion site, it has also been observed in cases with a history of surgery and residual cysts [7]. Several studies suggest that lesion growth follows a linear pattern, and rapid symptom progression may indicate malignant transformation [8].

Proposed risk factors for malignant transformation include long-standing, untreated cysts; incomplete prior resections; chronic inflammatory episodes (such as chemical meningitis due to cyst rupture); and advanced patient age, although a definitive causal relationship has not been established. Clinically suspicious signs include new or progressive neurological symptoms, changes in MRI characteristics, acute hydrocephalus, and slow growth of a previously stable lesion [9].

Radiologically, ECs usually present as low-intensity lesions within the subarachnoid space without contrast enhancement on CT or MRI and appear hyperintense on diffusion-weighted imaging (DWI) [10]. When transformation into SCC occurs, the lesions typically show significant contrast enhancement on CT and T1-weighted MRI. Peritumoral edema is observed as a secondary feature in approximately 50% of cases.

The reported interval between diagnosis of EC and transformation into SCC varies widely, ranging from six months to 33 years [11]. Surgical resection is rarely curative due to the involvement of vital neurovascular structures. However, postoperative radiotherapy has been associated with improved overall survival (OS), offering up to six additional months of life compared to surgery alone [11]. In one case series, postoperative radiotherapy following gross total resection yielded a one-year OS of 72.5%, with a median survival time of 24 months - the only factor found to significantly influence survival [12]. While immunotherapies such as immune checkpoint inhibitors have been approved for frontline treatment of SCC of the head and neck, optimal treatment combinations for SCC arising from ECs remain undefined [13].

This article was previously presented as a meeting abstract at the 2024 EANO Scientific Meeting on October 18, 2024.

## Case presentation

A 55-year-old female with a history of migraine was incidentally diagnosed at the age of 30 with a right CPA lesion suggestive of an EC. In March 2023, she presented with sudden-onset hearing loss in her right ear, accompanied by tinnitus, vertigo, headache, dysgeusia, and gait disturbances. Clinical examination revealed involvement of the right eighth cranial nerve, with findings consistent with sensorineural hearing loss, as well as complete right facial nerve palsy. A head MRI was performed, which revealed a lesion in the CPA with radiologic features concerning for malignancy. Given the patient’s history and classic symptoms (hearing loss, tinnitus, and headache), surgical intervention was pursued.

A right-sided retromastoid craniotomy using a retrosigmoid approach with intraoperative neurophysiological monitoring was performed. Intraoperatively, the lesion appeared partially friable with a calcified-like lining that was firmly adherent to the brainstem, middle cerebellar peduncle, and perforating vessels, rendering total resection unsafe. Despite maximal effort, brainstem auditory evoked potentials, blink reflex testing, and facial nerve conduction studies revealed abnormal results, consistent with axonal neuropathy of the right facial nerve. A ≥90% side-to-side difference in nerve conduction was observed, along with profound right-sided hearing loss. A contrast-enhanced MRI of the neuraxis showed no evidence of additional lesions.

**Figure 1:**
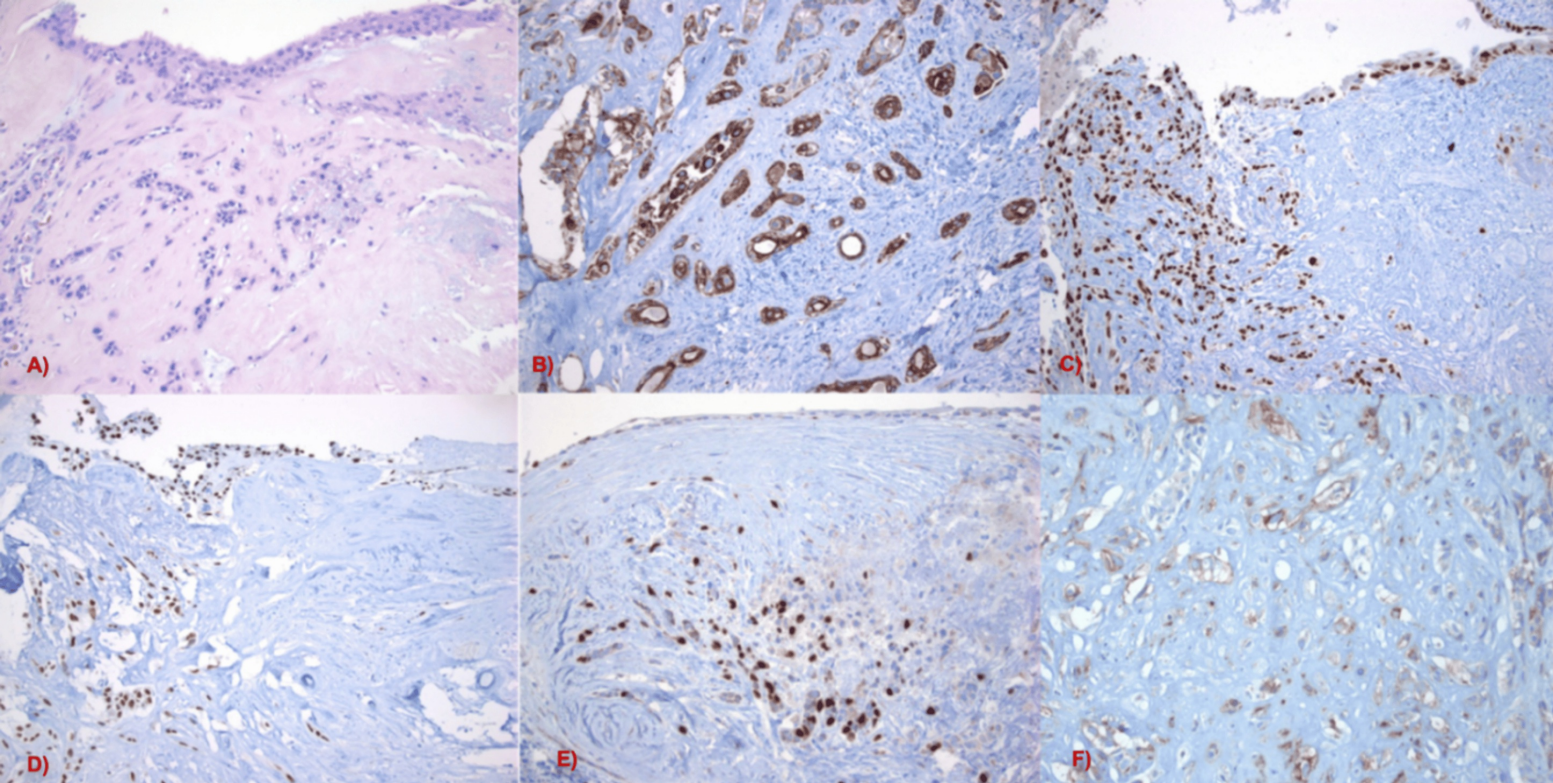
Histopathological features of the tumor Sections stained with H&E reveal a cystic cavity lined by stratified epithelial cells, with malignant cells (CA) infiltrating the underlying parenchyma (A). Both EC cells and SCC cells show positive immunoreactivity for CK5/6 (B), p40 (C), and p63 (D). Ki-67 (MIB1) staining demonstrates nuclear positivity in 20% of SCC cells (E). PD-L1 expression is observed in some malignant cells as well as in peritumoral inflammatory cells (F). EC, epidermoid cyst; SCC, squamous cell carcinoma

Histopathological analysis of H&E-stained sections revealed a cyst lined by cells with basophilic, round to ovoid nuclei and abundant eosinophilic cytoplasm. The cyst lining was stratified, with evidence of cellular proliferation and invasion into the surrounding parenchyma. The infiltrating cells demonstrated atypia and moderate pleomorphism. Immunohistochemistry showed that the tumor cells were positive for p63, p40, CK5/6, and D2-40, supporting squamous differentiation. The tumor cells were negative for brachyury, effectively ruling out chordoma as a differential diagnosis. The proliferation index, assessed by Ki-67 (MIB-1), was 20%. PD-L1 (SP263) staining was also performed to calculate the combined positive score, which was 12.5. The morphological and immunohistochemical features were consistent with SCC originating from an EC (Figure 1).

**Figure 2:**
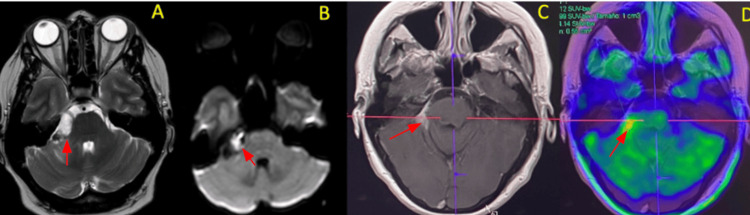
MRI and PET imaging (A, B) Initial MRI T2-weighted and DWI sequences (March 2023) revealed a right CPA lesion with heterogeneous enhancement, increased size, and extension into the internal auditory canal. (C, D) Contrast-enhanced MRI and ¹⁸F-FDG brain PET demonstrated focal hypermetabolism in the right CPA region, with a maximum standardized uptake value of 10.3. CPA, cerebellopontine angle; DWI, diffusion-weighted imaging

A one-month follow-up brain MRI showed a right CPA lesion with heterogeneous enhancement, increased diameter, and extension into the internal auditory canal. An ¹⁸F-FDG PET/CT demonstrated focal hypermetabolism in the right CPA, with a maximum standardized uptake value of 10.3, indicative of disease progression (Figure 2).

**Figure 3:**
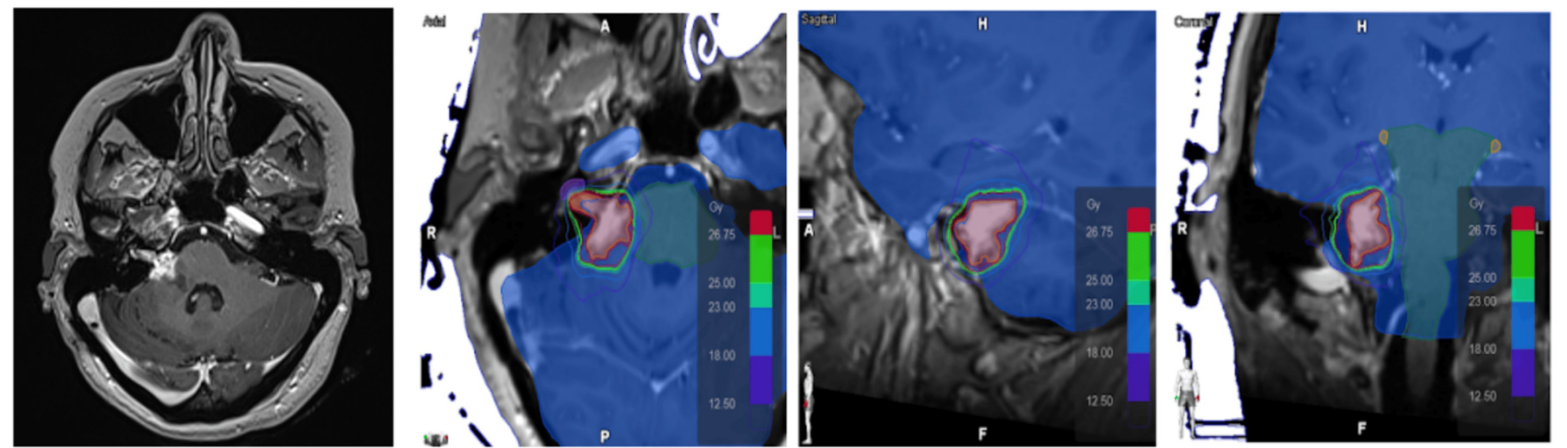
Three-dimensional dose distribution of fractionated stereotactic radiotherapy Axial, sagittal, and coronal sections show the target volume (tumor) in pink, surrounded by isodose lines representing various radiation dose levels, according to the color scale in gray (Gy). These images illustrate target coverage, treatment conformality, and dose distribution relative to adjacent anatomical structures. The treatment plan is designed to maximize tumor dose while minimizing exposure to surrounding healthy tissue.

She was treated with fractionated stereotactic radiotherapy to the residual lesion, receiving a total dose of 30 Gy in five fractions (6 Gy per fraction), prescribed to the periphery of the target volume. The treatment was delivered using a linear accelerator with non-coplanar volumetric arcs (Figure 3) and was completed on October 10, 2023.

A six-month follow-up ¹⁸F-FDG PET/CT showed no evidence of distant hypermetabolic activity, with persistent but decreased focal hypermetabolism in the right CPA compared to the previous scan (Figure 2). The patient completed six cycles of carboplatin, paclitaxel, and pembrolizumab, experiencing only mild grade 1 toxicities - alopecia and fatigue - and maintained stable disease status according to RECIST criteria for more than two years after diagnosis.

Although her facial nerve function and hearing did not recover, she reported an 80% improvement in tinnitus, vertigo, and headaches. Her overall quality of life remains good, and she is fully independent in all daily activities. She continues to undergo regular PET/CT imaging and multidisciplinary follow-up.

## Discussion

Primary SCC arising from an intracranial EC is exceptionally rare and presents a significant clinical challenge, especially for clinicians encountering CNS ECs for the first time. One of the most critical aspects of managing ECs lies in determining the optimal therapeutic approach and follow-up strategy, given the unpredictable potential for malignant transformation. The median latency period for transformation into SCC has been reported to be approximately 60 months; however, in patients who do not undergo surgical resection, this interval can be significantly shorter, sometimes as brief as 10 months. These findings emphasize the importance of long-term surveillance, particularly in cases where complete surgical resection is not achievable [10].

Early diagnosis remains challenging, particularly in populations with limited access to specialized healthcare services [14]. This issue affects not only the initial detection of ECs but also the ability to provide appropriate long-term follow-up to monitor for malignant changes. Neuroimaging is vital for diagnosis and surveillance. Typical EC findings include low signal intensity on T1-weighted MRI, high signal intensity on T2-weighted MRI, strong restriction on DWI, and lack of enhancement on gadolinium-enhanced sequences. In contrast, SCC generally demonstrates intense enhancement on gadolinium-enhanced T1-weighted images, heterogeneous signal on T2, and variable DWI restriction. Additional findings such as peritumoral edema, invasion into adjacent structures (e.g., internal auditory canal or brainstem), and necrotic regions may also be present, features not typically seen in benign ECs [15].

In our patient, the primary risk factors for malignant transformation included long-standing cyst duration (25 years) and chronic inflammation leading to epithelial dysplasia. Other risk factors reported in the literature, although not applicable in this case, include incomplete resection, prior radiation therapy, advanced age, and molecular alterations such as mutations in *p53 *or activation of the *EGFR *pathway.

Current treatment strategies primarily include microsurgery and radiotherapy. Radiation therapy has shown acceptable tumor control with relatively low recurrence rates, although it can be associated with hearing loss and cranial nerve dysfunction. Surgical resection, while potentially curative, is technically demanding due to the complex anatomy of the CPA. It aims for complete tumor removal and, in selected cases, hearing preservation. Surgical approaches include translabyrinthine, middle fossa, and retrosigmoid (suboccipital), with the choice depending on tumor size, canal involvement, baseline hearing, and surgeon experience. In our patient, a retrosigmoid approach was used; however, hearing preservation was not achieved. Known disadvantages of this approach include CSF leak, postoperative headache, and an increased risk of recurrence due to difficulty accessing the intracanalicular portion of the tumor [16].

As seen in our case, where the tumor was firmly adhered to neurovascular structures, subtotal resection was unavoidable. Similar cases of SCC arising in the CPA have shown tumor recurrence years later when remnants adhered to the brainstem were left in place [16]. This further supports the need for long-term follow-up, although defining an exact surveillance timeline is difficult given the variable latency of malignant transformation. Moreover, recurrence or transformation can occur at sites distant from the primary lesion, even post-resection [17].

Several reports of ECs located in the CPA have described significant cochlear symptoms, diplopia, and both motor and sensory disturbances of the trigeminal nerve [17], findings that are consistent with the present case. However, while these clinical manifestations are commonly observed, they lack specificity for distinguishing ECs from other tumor types in the CPA region. Although treatment strategies remain a topic of debate, surgical resection followed by postoperative stereotactic radiosurgery is emerging as a promising approach, showing improved outcomes for SCC arising in the CPA [18]. This multimodal treatment strategy was implemented in the present case, and the patient also demonstrated a favorable response to chemotherapy.

## Conclusions

This case highlights a rare but serious complication of a long-standing intracranial EC - malignant transformation into SCC. Despite its rarity, this possibility should be considered in patients with a history of EC who present with new or rapidly progressive neurological symptoms. The diagnostic and therapeutic challenges associated with this entity underscore the need for a multidisciplinary approach. Postoperative radiotherapy and immunotherapy may provide additional benefit, especially in cases where total resection is not feasible. Lifelong surveillance is strongly recommended due to the potential for delayed malignant transformation and recurrence.
